# Surgical options in the management of cystic duct avulsion during laparoscopic cholecystectomy

**DOI:** 10.1186/1754-9493-2-17

**Published:** 2008-06-20

**Authors:** Faramarz Karimian, Ali Aminian, Rasoul Mirsharifi, Farhad Mehrkhani

**Affiliations:** 1 Department of Surgery, Imam Khomeini Hospital Complex, Tehran University of Medical Sciences, Tehran, Iran

## Abstract

**Background:**

Avulsion of cystic duct during laparoscopic cholecystectomy (LC) is not a common intraoperative complication, but may be encountered by any laparoscopic surgeon. Surgeons are rarely familiar with management of this condition.

**Methods:**

Patients with gall stone related problems who were scheduled for LC at the minimal invasive surgery unit of a tertiary referral hospital during a 5 years period (April 2002–April 2007) were prospectively enrolled.

**Results:**

12 cases were identified (incidence: 1.15%). All 12 patients had gallbladder inflammation. Five patients had acute and seven patients had chronic cholecystitis. The avulsed cystic duct (ACD) was managed by clipping in 4, intracorporeal suturing in 3, converting to open surgery with suture ligation in 2, and lonely external drainage in 3 patients. Bile leakage had ceased within 3 days in 2, 14 days in one, and 20 days in the other patient. Bile volume increased gradually in one of the patients, which stopped only after endoscopic sphincterotomy (ES) at 25^th ^postoperative day. No major late complication or mortality occurred.

**Conclusion:**

ACD during LC is a rare complication. Almost all standard methods of treatment yield to successful outcomes with low morbidity. According to the situation, ACD may be successfully managed laparoscopically. Available cystic stump remnant was clipped. Intracorporeal suture ligation was performed when short length of stump precluded clipping. Deeply retracted cystic duct with active bile leak led to conversion to open surgery. With minimal or no bile leak at ACD stump, closed tube drainage of sub-hepatic area was attempted. Persistent bile leak was assumed to be controlled by ES, successfully accomplished in one patient.

## Background

Laparoscopic cholecystectomy (LC) is the most commonly performed operation of the digestive tract. Many reports have cited increased use of cholecystectomy after the introduction of laparoscopy [1]. Concomitantly, it has been well established that as LC was gaining popularity, the number of bile duct injuries increased [2-8]. In one statewide audit, the number of bile duct repairs was almost tripled between 1988 and 1992 [5]. It now appears that the rate of major injuries to the bile duct following LC is in the range of 0.2 to 0.4 percent, yielding 1000–2000 serious injuries in the USA [6].

LC usually begins with dissection at gallbladder neck to find cystic duct. In many instances this area is involved with inflammation and adhesion which obscure the anatomical details and predispose the cystic duct to injury during dissection. The applied traction to the gall bladder neck is a common surgical maneuver during LC. The traction force would be inevitably transferred to cystic duct and may be more than its tensile strength. Sometimes, after completing the skeletonizing of the duct, the surgeon forgets to reduce the traction force and therefore the duct, devoid of its supporting tissue, is avulsed at the weakest part. Acute inflammation (i.e. acute cholecystitis) may compromise microvascular circulation and lead to relative ischemia, rendering tissues to structural disintegration.

When faced with this technical complication, the surgeon is confronted with a challenging task. Cystic duct stump must be secured, injury to bile ducts must be avoided, and cholecystectomy should be performed. Management of avulsed cystic duct (ACD) during LC is the subject of this study.

## Methods

Patients with gall stone related problems who were scheduled for LC at the minimal invasive surgery unit of a tertiary referral hospital during a 5 years period (April 2002–April 2007) were prospectively enrolled. Nine surgeons were performing LC in our institution. The following parameters were recorded: age, sex, presence of acute or chronic inflammation at the time of operation, management of ACD stump, presence of minor bile leakage at the end of operation, placement of drain, presence and duration of postoperative bile flow through drain, second operation, post operative endoscopic sphinchterotomy (ES), and late complications with a minimum follow up of 6 months.

## Results

Of 1041 LCs performed during the study period, we encountered 12 cases of intraoperative ACD (incidence 1.15%). Nine surgeons had 12 cases, 6 had one case each and 3 had 2 cases.

Patients' data were summarized in table 1. There were 10 female and 2 male patients with a mean age of 48 (range 34–72) years. All 12 patients had gallbladder inflammation. Five patients had acute and seven had chronic cholecystitis. None of the patients underwent intra-operative cholangiography before avulsion of cystic duct. The ACD was managed by clipping in 4, intracorporeal suturing in 3, converting to open surgery with suture ligation in 2, and lonely external drainage in 3 patients. Those surgeons with more than one such case had not followed a similar technique in different patients.

**Table 1:** Characteristics of patients with ACD during LC

**N**	**Sex**	**Age**	**Diagnosis**	**Procedure**	**Leak at end of operation**	**Drain insertion**	**Duration of drainage**	ES*
1	F	41	Chronic cholecystitis	Clipping	No	No**	-	No
2	F	59	Chronic cholecystitis	Clipping	No	No	-	No
3	F	44	Chronic cholecystitis	Clipping	Yes	Yes	3 days	No
4	F	39	Acute cholecystitis	Clipping	Yes	Yes	3 days	No
5	F	47	Acute cholecystitis	Suturing	No	Yes	-	No
6	F	53	Acute cholecystitis	Suturing	No	Yes	-	No
7	F	37	Chronic cholecystitis	Suturing	No	No	-	No
8	F	39	Chronic cholecystitis	Open ligation	No	Yes	-	No
9	F	51	Acute cholecystitis	Open ligation	No	Yes	-	No
10	F	34	Acute cholecystitis	Drainage alone	No	Yes	20 days	No
11	M	61	Acute cholecystitis	Drainage alone	Yes	Yes	30 days	Yes
12	M	72	Acute cholecystitis	Drainage alone	Yes	Yes	14 days	No

*Endoscopic sphincterotomy.

**But she developed bile collection and underwent percutaneous drainage

Drain in sub-hepatic space was inserted in 9 patients. Minor bile leakage at the end of operation was present in 4 patients. Post operative bile leakage through drains was observed in 5 patients. Bile leakage had ceased within 3 days in 2, 14 days in one, and 20 days in the other patient. Bile volume which had increased gradually in one patient stopped only after ES at 25^th ^postoperative day. One of 3 patients in whom drain was not inserted intraoperatively, required percutaneous drainage of bile collection at 6^th ^postoperative day. None of the patients underwent reoperation. No late complication related to surgical injury to the bile ducts was observed in the follow up period.

## Discussion

Avulsion of cystic duct during cholecystectomy is not common, but may be encountered by any laparoscopic surgeon. Obviously, this usually happens during difficult operations, leading to further complications. Acute inflammation or chronic adhesion with perplexing dissection around cystic duct associated with excessive traction at gallbladder neck lead to the risk of ductal disintegration. Surgeons are rarely familiar with managing this condition. Four different approaches were chosen in our patients.

Many conditions may preclude safe access to the stump of an ACD, including acute inflammation and edema, chronic inflammation obscuring anatomical landmarks, a long cystic duct with a spiral course behind the common bile duct and a cystic duct running parallel and close to common bile duct. Also, the applied tension to the gallbladder neck may rotate the common bile duct around its long axis. This may hide the cystic duct behind it, when relaxed. Under these conditions, injudicious dissection along bile ducts can lead to major traumatic injury or may cause ischemia that subsequently presents as bile duct stricture.

Ideally, if adequate stump of the cystic duct is available, clipping is the first choice. But since the remnant of cystic duct is no longer tense and lengthy, applying one or two secure clips exactly perpendicular to its long axis may not be easily accomplished. Therefore, suboptimal clipping of cystic duct and subsequent bile leak may be expected. Clipping was achieved in only 4 patients. There was minor bile leak at the end of operation in two of them. Bile flow through drain had stopped within 3 days in both patients. The other 2 patients did not have bile leak at the end of the operation and drain was not placed for them. One of these patients developed a sub hepatic bile collection which was drained percutaneously. Bile leak after apparently successful application of clip to cystic duct remnant is presumably due to tangential application of the clip which left an opening at one corner.

Intracorporeal suturing is an advanced skill in laparoscopic surgery. Suturing the stump of an ACD is presumably a difficult task. The wall of normal size common bile duct which was buried in inflammatory tissue may be sutured spuriously, with the potential risk of late stricture formation. Two surgeons sutured the remnant of cystic duct in 3 patients. Postoperative bile flow through drains or bile collection were seen in none of the 3 patients.

Two patients were converted to open procedure. Cystic duct stumps showed active bile flow. They were retracted deep into inflammatory tissue and could not be safely secured by laparoscopy. Suture ligation was carried out on both patients during open surgery. No bile flow was detected through the drain during postoperative period.

The traditional response to a difficult LC is conversion to open procedure, but this may result in increased postoperative pain, delayed morbidity, adhesion formation and incisional hernia [8]. Therefore, laparoscopic subtotal cholecystectomy without cystic duct ligation has been suggested in these situations [8-10]. This procedure consists of resecting anterior wall of the gallbladder, removing all stones and placing a drain into Hartmann's pouch. Approximately 20% of patients developed postoperative bile leakage and some of them underwent ES with or without stent placement [8]. According to these studies, drainage alone may also be a safe option for cystic duct avulsion. In this study, ACD remnant could not be located in 3 patients (one female and two males) with acute cholecystitis. They were managed only by placement of drain in sub-hepatic fossa. Postoperative bile flow through drain stopped after 20 days in female patient. Minor bile leak was present in both male patients at the end of the operation. Both were elderly patients with associated comorbidities. Their surgeons decided not to proceed with any further action. Bile flow stopped through the drain after 14 days in one patient. In the other patient, the volume of the bile drainage increased. This patient underwent ES at 25^th ^postoperative day. The bile flow decreased gradually and stopped within a few days. None of these patients showed any sign of uncontrolled sepsis that indicated localized or diffuse peritonitis mandating surgical intervention.

Several studies have showed that "postoperative" cystic duct leak is a rare complication of LC and associated with fairly low morbidity. Endoscopic ductal decompression and percutaneous drainage are effective treatment [11-13]. We described that ACD may be managed with several techniques. Similarly, when persistent bile leakage occurred, ES was also effective.

## Conclusion

ACD during LC is a rare complication. Almost all standard methods of treatment yield successful outcome with low morbidity. This is best achieved by intracorporeal or open suture ligation of the stump. All patients should have a drain placed at sub-hepatic space. ES should be considered in persistent postoperative bile drainage.

## List of abbreviations

LC: laparoscopic cholecystectomy; ACD: avulsed cystic duct; ES: endoscopic sphinchterotomy.

## Competing interests

The authors declare that they have no competing interests.

## Authors' contributions

FK participated in the design of the study and drafted the manuscript, AA revised the paper and prepared it for publication, RM supervised the team and participated in the revision of the paper, FM collected the data and helped to draft the manuscript. All authors read and approved the final manuscript.
